# MALVIRUS: an integrated application for viral variant analysis

**DOI:** 10.1186/s12859-022-04668-0

**Published:** 2022-04-19

**Authors:** Simone Ciccolella, Luca Denti, Paola Bonizzoni, Gianluca Della Vedova, Yuri Pirola, Marco Previtali

**Affiliations:** 1 Department of Informatics, Systems and Communication, University of Milano-Bicocca, Viale Sarca 336, 20136 Milan, Italy; 2 Department of Computational Biology, Institut Pasteur, 25-28 Rue du Dr Roux, 75015 Paris, France

**Keywords:** Sequence analysis, Genotyping, Lineage classification, Virus, SARS-CoV-2

## Abstract

**Background:**

Being able to efficiently call variants from the increasing amount of sequencing data daily produced from multiple viral strains is of the utmost importance, as demonstrated during the COVID-19 pandemic, in order to track the spread of the viral strains across the globe.

**Results:**

We present MALVIRUS, an easy-to-install and easy-to-use application that assists users in multiple tasks required for the analysis of a viral population, such as the SARS-CoV-2. MALVIRUS allows to: (1) construct a variant catalog consisting in a set of variations (SNPs/indels) from the population sequences, (2) efficiently genotype and annotate variants of the catalog supported by a read sample, and (3) when the considered viral species is the SARS-CoV-2, assign the input sample to the most likely Pango lineages using the genotyped variations.

**Conclusions:**

Tests on Illumina and Nanopore samples proved the efficiency and the effectiveness of MALVIRUS in analyzing SARS-CoV-2 strain samples with respect to publicly available data provided by NCBI and the more complete dataset provided by GISAID. A comparison with state-of-the-art tools showed that MALVIRUS is always more precise and often have a better recall.

**Supplementary Information:**

The online version contains supplementary material available at 10.1186/s12859-022-04668-0.

## Introduction

The SARS-CoV-2 pandemic has put the global health care services to the test and many researchers are racing to face its swift and rapid spread. Since the outbreak of the virus in China and in other European countries, several studies are using sequencing technologies to track the geographical origin of SARS-Cov-2 and to analyze the evolution of sequence variants [1–3] or to understand the role played by human genes on viral replication [4–7]. In this context, the availability of efficient approaches to analyze variations from the growing amount of sequencing data daily produced is of the utmost importance.

The typical pipelines for the analysis of variations in viral samples consists of aligning reads against a reference genome [8], then analyzing the alignments to discover the variants [9, 10]. However, the increasing number of viral assemblies available in public databases such as GISAID [11], GenBank [12], and the COVID-19 Data Portal allows to build a complete catalog of variants of a viral population. Such a catalog can be used to reduce the complexity of comparative analysis of genetic variants of sequencing samples. For this goal, it is crucial that users are assisted by an efficient and easy-to-use method for building and updating the catalog and for calling and annotating variants that are in this catalog. In this paper, we introduce MALVIRUS, a web application to quickly analyze newly sequenced viral read samples, including—but not only limited to—SARS-CoV-2 samples. Particularly for this novel virus, more and more interest is given to the different Pango lineages of the virus [3] (from now on we implicitly assume that lineages are referred to the Pango nomenclature) since different viral lineages exhibit different levels of infection rate and virulence [13, 14]. For this reason, MALVIRUS allows for determining the lineage from which a read sample originates—the so called lineage assignment problem—using the well-known tool pangolin [15] directly from the read sample without assembling the full-length genome. This step is especially relevant since some lineages are classified by major health organizations as Variants Of Concern (VOC) or Variants of Interest (VOI) [16] due to their peculiar characteristics (for example, because they may exhibit resistance to vaccines [17, 18]) that suggest an emerging risk to global public health and, hence, their spread should be attentively monitored and tracked.

We evaluated MALVIRUS accuracy in genotyping, annotating, and classifying newly sequenced SARS-CoV-2 strains on both short and long read data. Since MALVIRUS heavily depends on the current knowledge available (i.e., the set of variations characterizing the population under investigation), we also propose and test different methodologies for building the variant catalog. The proposed pipelines are freely available and can be used with any set of assemblies. In our experimental evaluation, we considered catalogs built on the set of SARS-CoV-2 assemblies freely available on NCBI as well as on the ones available from GISAID. However, we believe that thanks to our pipelines, implemented as Snakemake workflows [19], any user can easily build a variant catalog starting from his own private set of assemblies.

MALVIRUS is distributed as a multi-platform Docker container [20] and it can be easily accessed using any modern Internet browser.

## Implementation

MALVIRUS is a user-friendly application for efficiently genotyping a viral sample. MALVIRUS is based on MALVA [21] and builds around it a complete and user-friendly infrastructure of scripts and pipelines to facilitate the genotyping of viral samples. MALVA is an efficient tool for genotyping a sample with respect to a catalog of variants without mapping the reads to the reference genome. To assist the user, MALVIRUS provides both a set of precomputed variant catalogs periodically generated from the publicly available SARS-CoV-2 genome assemblies and the ability to compute a variant catalog from a set of user-provided genome assemblies (not necessarily SARS-CoV-2). Furthermore, MALVIRUS assists the user in performing the genotyping task and in visualising the result. For SARS-CoV-2 samples, MALVIRUS also performs the functional annotation of the reconstructed genotype and the Pango lineage assignment of the sample. A Pango lineage [3] is a cluster of SARS-CoV-2 sequences associated with an epidemiological event.

The rest of this section describes the methods related to catalog creation and presents the pipeline for genotyping the read sample.

### Precomputed catalogs

As already stated, MALVIRUS genotypes a set of variations contained in a catalog, representing the current state of the art on all known variants observed in the population of interest. For user convenience, MALVIRUS is distributed with a set of precomputed catalogs for the SARS-CoV-2 viral genome, thus users can immediately run MALVIRUS on a locally available (e.g., private) viral sample. Moreover, the precomputed catalogs can be easily updated from the application itself with a single click.

**Fig. 1:**
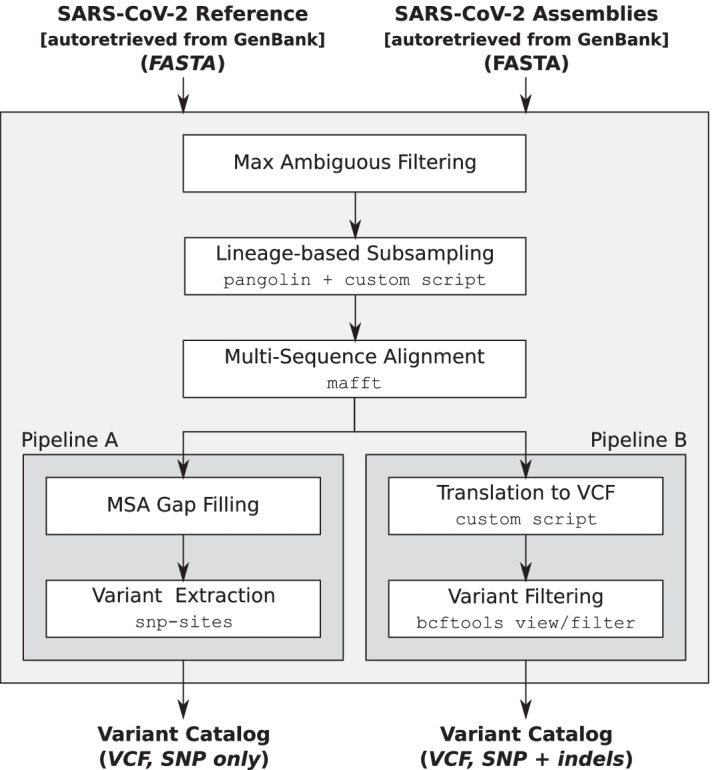
Precomputed catalog creation. Schematic representation of the pipelines used to create the precomputed SARS-CoV-2 catalogs from the set of public assemblies available on GenBank

The primary aim of the precomputed catalogs is to ensure that the variations they contain represent as completely as possible the set of sequence variations that are present in SARS-CoV-2 genomes sequenced to date, all while maintaining high quality (in order to avoid including variations deriving from technical artifacts) and small size (in order to keep the tool efficient). To achieve this twofold aim, we specifically designed a pipeline that extracts variants from the set of all the assemblies available on GenBank [12]. Figure 1 depicts the main steps of the pipeline.

The set of assemblies is initially preprocessed by filtering out sequences with more than $\tau _{N} = 1\%$ ambiguous nucleotides, then the resulting set is subsampled based on their Pango lineage [3]. Subsampling is performed by assigning the lineage to each assembly using pangolin [15], then by keeping 1% of the sequences assigned to each lineage with a minimum of $min_{lin}$ and a maximum of $max_{lin}$ sequences for each lineage. If a lineage has less than $min_{lin}$ sequences, then all its sequences are kept. Parameters $min_{lin}$ and $max_{lin}$ are chosen in order to provide the best trade-off between completeness of the catalogs and computational efficiency of the tool. Currently they are set to 50 and 100, respectively, but their values are subject to change in future updates depending on the number (and the quality) of genome assemblies deposited to GenBank.

Due to the importance of lineages classified as Variants Of Concern/Interest (VOC/VOI) for epidemiology [16] and to ensure that they are sufficiently represented in the precomputed catalogs, the parameters $min_{lin}$ and $max_{lin}$ for those lineages are increased to $5\times min_{lin}$ and $5\times max_{lin}$, respectively. Currently, we increased the two parameters for lineages *B.1.1.7*, *B.1.351*, *P.1*, *A.23.1*, and *B.1.525*, that are the subjects of the cov-lineages.org global reports [22]. Table 1 summarizes the frequency distribution of lineages in GenBank as of April 7, 2021 and provides the indication of the most represented lineages (left) and of VOC/VOIs (right) in our precomputed catalogs. As an example of how sequences were sampled, on April 7, 2021, there were 9390 sequences assigned to lineage *B.1* (over a total of 78,098 sequences that passed max ambiguous nucleotides filtering), hence only 93 of them were randomly selected to create the catalog. As another example, there were 16,844 sequences assigned to lineage *B.1.2* and, since the 1% of 16,844 is greater than $max_{lin} = 100$, only 100 of them were selected to create the catalog. On the other hand, there were 1835 sequences assigned to lineage *A.1* and, since the 1% of 1835 is less than $min_{lin} = 50$, 50 of them were selected to create the catalog.

**Table 1:**
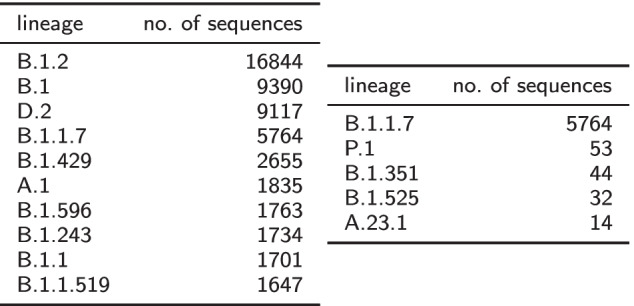
GenBank assemblies information

Number of assemblies deposited on GenBank for the most represented lineages (left) and for 5 Variants of Concern (right). Lineages were assigned using pangolin. A total of 626 lineages are present, but 125 of them (about 20%) have only a single sequence and 56 (about 9%) have only two sequences. Sequences were retrieved on April 7, 2021

Two catalogs are generated from the preprocessed and sampled sequences. The first one only considers SNPs, while the second one also considers indels of length up to 10nt. The first catalog is created using a pipeline (called Pipeline A, from now on) that first builds the multiple sequence alignment of the sampled full-length sequences to the viral reference genome (NCBI Reference Sequence: NC_045512.2) using MAFFT [23], and then extracts the set of population SNP loci from the multiple alignment using snp-sites [24]. Since snp-sites is not able to output variations in positions with gaps, gaps of the alignment are filled with the corresponding portions of the reference. As a consequence, indels are not present in the resulting catalog, and this should lessen the impact of technical (or computational) artifacts that are present in the deposited sequences. For example, sequences LR897977.1-LR898047.1 have one-base deletions approximately at every 60 bases and these deletions would introduce likely false variations in the catalog.

The second catalog is created using a pipeline (called Pipeline B, from now on) that, as Pipeline A, uses MAFFT to build the multiple alignment of the sampled sequences, but then uses an in-house script to translate the alignment to a set of sequence variations stored in a VCF file.

Differently from snp-sites, our custom script is also able to extract indels, that, besides SNPs, apparently have a role in determining the characteristics of the virus. For example, the spike deletion 69-70del has been described in the context of evasion to the human immune response [25]. On the other hand, the inclusion of indels might introduce some false variations due to technical artifacts in the input assemblies.

*Custom catalogs.* If the user wants a finer control over the variant catalog or if she/he wants to use a private set of assemblies (that cannot be freely shared), MALVIRUS interface allows to create a custom catalog from a set of assemblies or to directly upload a catalog in VCF format.

For the automatic creation of the catalog starting from a set of assemblies, MALVIRUS requires as input the reference genome of the viral species under investigation (for example, to study species different from SARS-CoV-2), the set of assemblies, and, if available, the annotation of the genes. Then, the catalog is created using Pipeline A (as previously described) without subsampling the sequences based on their assigned lineages. The rationale is that, in this case, we do not want to interfere with selection process performed by the user.

In any case, although MALVIRUS interface does not directly allow it, a user can create a catalog using Pipeline A or B (that we freely distribute) and upload the VCF file through MALVIRUS interface.

### Variant genotyping and lineage assignment

**Fig. 2:**
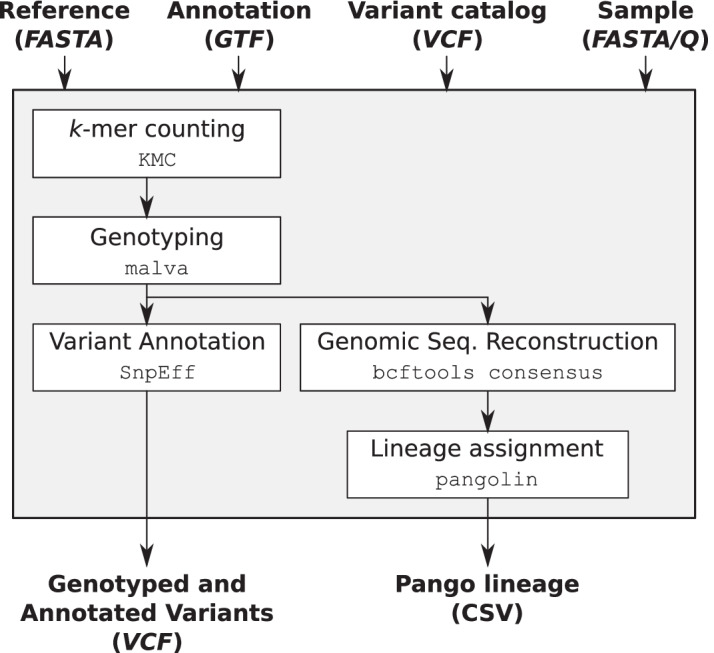
Variant genotyping and lineage assignment. Schematic representation of the variant genotyping and lineage assignment module of MALVIRUS

MALVIRUS allows to genotype a newly-sequenced sample and assign it to its most likely Pango lineage [3]. For this task, MALVIRUS requires as input a sample of reads in FASTA/Q format and a catalog of known variations (either created from a set of assemblies, uploaded, or chosen from the set of precomputed catalogs). The output of MALVIRUS consists of a VCF file containing the genotyped and annotated variations of the considered catalog and the most likely lineage assigned to the input sample. To fulfill these tasks, MALVIRUS integrates a pipeline (see Fig. 2) based on 5 state-of-the-art tools: KMC, MALVA, SnpEff, BCFtools, and pangolin.

First of all, MALVIRUS genotypes the input variants using MALVA [21], an efficient and accurate mapping-free approach for genotyping a set of known SNPs and indels, initially developed for genotyping human individuals. MALVIRUS first counts $k$-mers in the sample using KMC [26], then genotypes with MALVA each input variant (i.e., each variant in the input catalog) exploiting $k$-mers frequencies and using a multinomial probability model to take into account multi-allelic variation, that are those variations with more than one alternate allele (a common situation with population VCFs). Since MALVA was initially developed for human individuals, before integrating it in MALVIRUS, we extended it to support haploid organisms, such as viruses. Moreover, MALVA was originally developed for Illumina short reads whereas most RNA viruses are sequenced using third-generation sequencing technologies (like Oxford Nanopore). Such samples exhibit a very high coverage since the viruses’ genomes are quite short. Thus, we modified MALVA to work with very-high-coverage samples.

Next, if gene annotation is available, MALVIRUS also annotates the functional effects of each genotyped variation using SnpEff [27]. This tool annotates a set of variations based on their reference position and predicts their functional effects on known genes. Variant annotation is relevant since it may help in shedding more light on the evolution of the considered genome [27].

Finally, MALVIRUS computes the most likely Pango lineage using pangolin [15]. Since pangolin only accepts full-length assemblies as input, we use BCFtools (more precisely, its *consensus* command) to reconstruct the genome sequence of the computed genotype. Please notice that the genome built in this step should not be considered as the complete genomic sequence of the sample as it is built with respect to only the variations that are present in the chosen catalog. pangolin uses a decision tree trained using SARS-CoV-2 GISAID sequences to assign a new sequence to a lineage, i.e., a cluster of sequences associated with an epidemiological event. Such assignment is of the utmost importance to better understand the expanding phylogenetic diversity of SARS-CoV-2 and to track its global spread [3].

**Fig. 3:**
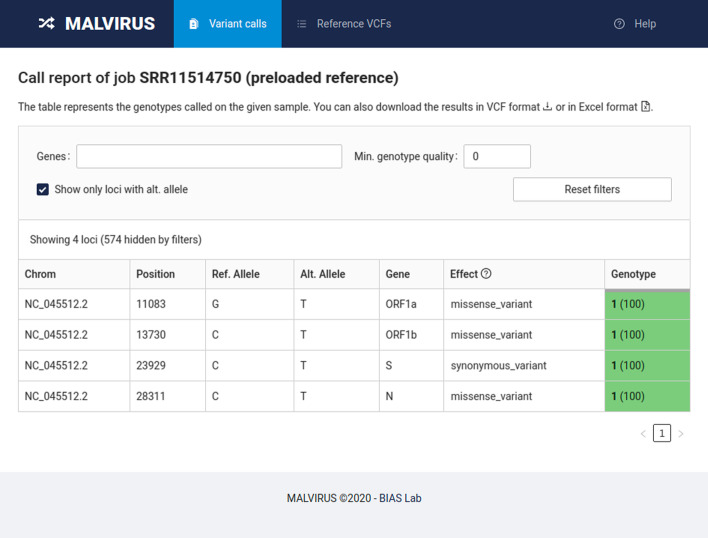
Report. Example of the final report of MALVIRUS

Finally, the results of each analysis can be visualized as a table (see Fig. 3 for an example) or downloaded in VCF format or as a spreadsheet for further analysis.

### Additional implementation details

MALVIRUS is available as a self-hosted web application distributed as a Docker container image that can be installed and run on multiple platforms, from personal laptops to large cloud infrastructures. All pipelines described in this section have been implemented as Snakemake workflows [19], thus easy to use and fully reproducible. Extensive documentation and a detailed tutorial are available at https://algolab.github.io/MALVIRUS.

## Results

To test the effectiveness of MALVIRUS, we performed two experimental evaluations. In the first one, we evaluated MALVIRUS accuracy in genotyping viral samples exploiting the current knowledge publicly available on the variants of SARS-CoV-2. Indeed, in this first analysis, we ran MALVIRUS using the precomputed catalogs distributed along with the application—which are also readily available to any user. The results of this first experiment should reflect the most common scenario a user may come across: genotyping and analyzing a new sample in the fastest and most straightforward way, without worrying about building an ad-hoc variation catalog.

In the second experimental evaluation, instead, we evaluated the accuracy of MALVIRUS pipeline in assigning the correct lineage to a viral sample. To better evaluate its accuracy, we created a variation catalog starting from the SARS-CoV-2 sequences available on GISAID (accessed on April 7, 2021). This experiment should reflect the more complex scenario in which a user wants to analyze a sample with respect to a given population of sequences of interest; for example a set of genomes that cannot be freely shared—such as GISAID itself—or a set of already available private samples for which a custom catalog needs to be generated.

**Table 2:** Samples considered in the experimental evaluation

SRA ID	Technology	Coverage	GISAID ID	Lineage
ERR5026409	ILL	62	730750	A.23.1
ERR5040238	ILL	725	756390	A.23.1
ERR5053597	ILL	366	768932	A.23.1
ERR5174710	ILL	753	858801	A.23.1
ERR5189961	ILL	707	862989	A.23.1
ERR5074718	ILL	123	706608	AP.1
ERR4082432	ONT	115	425449	B.1
ERR4246852	ONT	285	457212	B.1.1
ERR4437884	ILL	2200	489377	B.1.1.134
ERR4584869	ILL	1600	532794	B.1.1.253
ERR4668432	ILL	483	575675	B.1.1.255
ERR4615778	ILL	847	539900	B.1.1.269
ERR4439830	ILL	17	499533	B.1.1.323
ERR4438180	ILL	367	488906	B.1.157
ERR4848290	ILL	509	643640	B.1.160
ERR5011401	ILL	838	710801	B.1.1.7
ERR5042696	ILL	1000	760798	B.1.1.7
ERR5052852	ILL	457	769604	B.1.1.7
ERR5183522	ILL	726	846539	B.1.1.7
ERR5184915	ILL	854	833702	B.1.1.7
ERR4759202	ILL	105	595464	B.1.177
ERR4860691	ILL	401	646554	B.1.177.19
ERR5049949	ILL	804	767261	B.1.177.8
ERR5082229	ONT	136	680109	B.1.258.5
ERR5041219	ILL	815	762499	B.1.351
ERR5074602	ONT	27	764231	B.1.351
ERR5093255	ILL	226	819798	B.1.351
ERR5178844	ILL	141	812064	B.1.351
ERR5179824	ILL	631	821134	B.1.351
ERR4651735	ILL	430	567575	B.1.36.17
SRR13261896	ILL	10	708631	B.1.366
ERR4973704	ILL	467	655669	B.1.36.9
SRR13606385	ILL	55	903433	B.1.427
ERR5042239	ILL	983	760883	B.1.525
ERR5176822	ILL	459	797195	B.1.525
ERR5181246	ILL	564	836880	B.1.525
ERR5181360	ILL	633	836839	B.1.525
ERR5190001	ILL	89	863189	B.1.525
ERR4366428	ONT	13	493559	B.23
SRR11494747	ILL	186	419918	B.31
SRR13530301	ILL	58	873257	P.1

For each sample, we report the SRA Accession ID, the technology used (ILLumina or Oxford Nanopore Techonology), the coverage in terms of number of bases (in millions), the corresponding GISAID Accession ID (for ease of presentation we removed the *EPI_ISL_* prefix), and the Pango lineage computed using pangolin on the corresponding assembly downloaded from GISAID

For both experimental evaluations we randomly selected a total of 41 samples with raw reads available on the Sequence Read Archive (SRA) and that were also cross-referenced on the GISAID database (see Table 2 for the list of samples). In particular, we specifically included 5 randomly-selected samples of the lineages *B.1.1.7*, *B.1.351*, *P.1*, *A.23.1*, and *B.1.525* since these lineages are considered as Variants of Concern due to their peculiar epidemiological characteristics. For lineage *P.1* we were able to include only a single sample since we were not able to find other read samples on SRA cross-referenced on GISAID and assigned to that lineage. To balance the chosen set of samples, we included 20 samples assigned to other lineages (without stratification). Furthermore, the selected set includes samples sequenced using either Illumina or Oxford Nanopore Technologies in order to assess the accuracy of MALVIRUS on both technologies.

All the experiments can be reproduced (for the second experimental evaluation, after non-freely redistributable data are downloaded from GISAID) using the Snakemake workflows available at https://github.com/AlgoLab/MALVIRUS-repro/.

### Genotyping accuracy

In this part we assess the accuracy of MALVIRUS in genotyping a read sample on the precomputed catalogs that are readily available to the users and that have been computed as described in the Implementation section. We recall that the precomputed catalogs are built starting from all the SARS-CoV-2 genomic sequences deposited on GenBank, that are then filtered and subsampled according to their lineage and finally aligned to the reference. From the alignment, two catalogs of variants are computed using the pipelines A (on SNPs only) and B (on both SNPs and indels) as described before. To better evaluate how the choice of the subsampling parameters $min_{lin}$ and $max_{lin}$ affects the accuracy, we ran the two pipelines with three different combinations of the parameters value: the default setting $min_{lin} = 50$ and $max_{lin} = 100$, a more stringent setting $min_{lin} = 20$ and $max_{lin} = 50$, and a more relaxed setting $min_{lin} = max_{lin} = 100$. As such, a total of 6 different catalogs were generated and considered in this part.

We compared the accuracy of MALVIRUS with that of two state-of-the-art callers: BCFtools and lofreq (as indicated in most of the workflows published on Galaxy). Differently from MALVIRUS, these tools rely on the alignments of the input read sample to the reference genome to call variations. We aligned Illumina samples using BWA [28] whereas we mapped ONT samples with minimap2 [8].

Accuracy has been evaluated in terms of precision and recall and we considered as ground truth the variations induced by the alignment to the SARS-CoV-2 reference of the full-length genomic assembly associated to each sample as deposited to GISAID. The “true” genotype of each sample has been extracted from the alignment with the script that we use in the pipeline B for the construction of the precomputed catalogs. We classified each variant of the considered catalog as a *reference variant* if its true genotype is 0, i.e., the reference allele, and as an *alternate variant* if its genotype is not 0. Finally, we computed precision and recall of the tools in genotyping alternate variants: any alternate variant called alternate is considered a true positive; any alternate variant called reference is a false negative; and finally any reference variant called alternate is a false positive.

We report the results of the analysis in Table 3. MALVIRUS scored the best in terms of *precision* in all settings with an average of 96% against 91% and 76% of BCFtools and lofreq respectively; in terms of *recall* the tools score much closer with another clear advantage of MALVIRUS (94%) over the others (92% and 84%).

**Table 3:** Results on NCBI catalogs

Pipeline	$min_{lin}$	$max_{lin}$	MALVIRUS		bcftools		lofreq	
			Precision	Recall	Precision	Recall	Precision	Recall
A	20	50	**.951**	.919	.909	**.932**	.778	.856
B	20	50	**.972**	**.955**	.921	.909	.788	.823
A	50	100	**.951**	.924	.907	**.931**	.757	.856
B	50	100	**.967**	**.959**	.916	.909	.763	.821
A	100	100	**.952**	.924	.908	**.932**	.738	.857
B	100	100	**.968**	**.962**	.916	.909	.744	.822

For each catalog, we report the precision and recall achieved by MALVIRUS, BCFtools, and lofreq in calling the variations available in the catalog. The results are shown in terms of average over all the 41 considered samples. We highlighted in bold the best results. We considered 6 different catalogs built using pipeline A or B on the set of assemblies retrieved from NCBI, prefiltered using $\tau _N = 1\%$ and then subsampled using different combinations of parameters $min_{lin}$ and $max_{lin}$

We also analyzed the role of sequencing technology on the accuracy of predictions (Table 4). Using the default catalog (including indels), MALVIRUS achieved average precision of 99% and recall of 96% on the considered Illumina samples; whereas it scored 79% precision and 95% recall on the ONT ones. The change in sequencing technology does not affect MALVIRUS recall while it impacts its precision: this is mainly due to the higher error-rate of the latter type of data. Indeed, the same trend is also present in BCFtools (precision drops from 94 to 73%) and lofreq (77–68%). Notice that MALVIRUS is the tool with best precision on both types of sequencing technology. However, the gain in terms of precision with respect to alignment-based approaches was expected. Indeed, especially with exhaustive and complete input knowledge, genotyping a set of known variations is more precise than discovering variants from alignments [21].

**Table 4:** Results on NCBI catalogs depending on sequencing technology

Technology	MALVIRUS		bcftools		lofreq	
	Precision	Recall	Precision	Recall	Precision	Recall
ILL	**.991**	**.961**	.942	.899	.775	.817
ONT	**.792**	.946	.731	**.980**	.680	.845

We report the precision and recall achieved by MALVIRUS, BCFtools, and lofreq in calling the variations available in the default catalog (NCBI catalog, Pipeline B, $min_{lin} = 50$, $max_{lin} = 100$). The results are shown in terms of average over all the considered samples aggregated by sequencing technology. We highlighted in bold the best results

We note that lofreq is way less precise than the other tools. Indeed, analyzing its calls, we observed that lofreq calls a lot of variation with low quality: a post-filtering of its calls may improve its precision while affecting its recall (that is already lower than that of other tested approaches). Moreover, although alignment-based approaches do not rely on an input catalog of known variations, their accuracy varies with respect to the considered catalog since the set of variations of interest (i.e., the truth used to compute precision and recall) changes.

Finally, it is interesting to notice that increasing the quantity of sequences in the catalog creation has very little effect, while changing from pipeline A to B—thus including indels—produce a noticeable improvement on both precision and recall. It is important to remark that catalogs that include indels may also contain variants that are called only due to the presence of technical artifacts in the deposited assemblies, hence false variants. On the other hand, some deletions apparently have a role in determining the characteristics of the virus (for example, the spike deletion 69-70del has been described in the context of evasion to the human immune response [25]) hence, for some analyses, it is important to characterize and identify them. The improvement on both precision and recall using the catalogs containing indels provides an indirect evidence that MALVA’s predictions are robust against possible technical artifacts, but we believe that the ultimate choice of the catalog is upon the user based on the intended usage of MALVIRUS’s results.

On the default catalogs (those with $min_{lin} = 50$ and $max_{lin} = 100$), MALVIRUS, ran with a single thread, completed the analysis of each sample in 168 s on average. On the more stringent catalog ($min_{lin} = 20$ and $max_{lin} = 50$) it took 64 s while on the more relaxed one ($min_{lin} = 100$ and $max_{lin} = 100$), 410 s. This was expected since MALVIRUS execution heavily depends on the size of the input catalog: the more exhaustive and complete it is, the more computational time is required to analyze it.

In terms of memory requirements, MALVIRUS required less than 4GB of RAM in any tested settings. Such amount of resources is nowadays available on any computer, allowing MALVIRUS to run even on laptops and desktop machines. The computational requirements of catalog creation is dominated by the multiple alignment step. However, since these catalogs are precomputed and bundled with the application, this step is not executed on the user’s computer.

To complete the analysis of MALVIRUS results, we also analyzed how many samples have been assigned to the correct Pango lineage. MALVIRUS classified correctly 36–38 (over 41) of them (depending on the considered catalog, 36 for the smallest catalogs and 37–38 for the largest ones). MALVIRUS could not correctly classify all the samples since the set of assemblies currently freely available on NCBI does not contain a sufficient amount of information (i.e., enough assemblies) for correct lineage inference. In the following section, we thus further explore MALVIRUS effectiveness in inferring a lineage from a sample by considering a more complete and exhaustive (but not redistributable) database, i.e., GISAID.

### Lineage assignment accuracy

The second experiment has the goal to assess the quality of MALVIRUS in the assignment of SARS-CoV-2 lineages. In order to build a catalog as representative as possible of the different lineages, in this part we relied on not redistributable sequences from GISAID data (Additional file 1). We constructed a total of 8 catalogs from these sequences using the two previously described pipelines (A and B)—all reproducible with our published procedures after obtaining the data from the database, since those data cannot be shared. We used this platform because it currently (as of April 7, 2021) contains around 1 million of assembled sequences—in contrast to the approximately 70 thousands in NCBI/ENA—thus including a much high number of lineages.

In more detail, we downloaded all complete sequences available on GISAID (accessed on April 7, 2021) and we filtered out all those sequences having more than 5% ($\tau _N$ parameter) ambiguous nucleotides. From the remaining set of assemblies, we randomly selected either 5 to 20 or exactly 50 samples from each lineage and computed VCF catalogs according to the pipelines A and B. Then we pruned variants that had less than either 2 or 5 sequences supporting them to reduce the noise and errors contained in the large GISAID dataset. The different choice of the parameters $\tau _N$, $min_{lin}$, $max_{lin}$ and the addition of a final pruning step, compared to the procedure used to build the precomputed catalogs from publicly available data, is motivated by the significant difference in the number of sequences involved. Indeed, we increased $\tau _N$ from 1 to 5% in order to avoid discarding upfront lineages represented by a small number of sequences. At the same time, we have been more restrictive on subsampling (i.e., we reduced the minimum and the maximum number of sequences selected for each lineage) since, if all lineages are represented in the set of sequences, then a small number of sequences per lineage should suffice to include variants that characterize the lineage into the catalog. Furthermore, please notice that the additional pruning step we introduced should not filter out variants that are common in a lineage since the minimum support we require (2 or 5) is not greater than the minimum number of sequences we select for each lineage.

**Table 5:** Results on GISAID catalogs

Pipeline	$min_{lin}$	$max_{lin}$	Min support	Precision	Recall	Time (s)	No. of correct lineages
A	5	20	2	.953	.947	38	40
A	5	20	5	.951	.945	19	40
B	5	20	2	.992	.967	48	40
B	5	20	5	.993	.960	21	40
A	50	50	2	.933	.918	897	40
A	50	50	5	.942	.948	355	40
B	50	50	2	.960	.962	2465	40
B	50	50	5	.972	.960	677	40

For each catalog, we report the precision and recall achieved by MALVIRUS in genotyping its variations, the average running times, and the number of input samples (out of 41) assigned to the correct lineage. We considered 8 different catalogs, built using pipeline A or B on the set of assemblies retrieved from GISAID, prefiltered using $\tau _N = 5\%$ and then subsampled using different combinations of parameters $min_{lin}$ and $max_{lin}$. In addition, we also filtered out from the catalogs all variations present in less than either 2 or 5 assemblies (*Min support* columns)

As reported in Table 5, MALVIRUS consistently and accurately detects 40 out of 41 samples (see Table 2) achieving an accuracy of 97.5% on each of the 8 catalogs considered.

MALVIRUS pipeline always failed to correctly classify Illumina sample SRR13261896 (real lineage: B.1.366, USA lineage). We believe that MALVIRUS pipeline is unable to classify it correctly due to the low coverage of the Illumina sample. Indeed, as we can see from Table 2, SRR13261896 is the sample with the lowest coverage: this low coverage may make harder the genotyping process and the subsequent lineage assignment based on genotyped variations. Moreover, MALVIRUS assign this sample to lineage B.1.612, another USA lineage: since both these lineages are quite rare and come from the same region, we believe that it may be easier to mistake one for the other one.

This analysis allowed us to better evaluate the effect of the amount of samples needed for the catalog creation and the minimum allele support. Interestingly they both have very little effect on the precision and recall of variants detection ($\pm 2\%$), while increasing the number of support from 2 to 5 significantly reduces running times (66% on average) at none or negligible changes of the other scores.

Such results would suggest that increasing the number of samples used to build the catalogs will not yield measurable advantages in terms of precision and will on contrary worsen running times, due to the higher number of variations; thus we suggest using pipeline B with parameters $min_{lin} =5$, $max_{lin} =20$ and with a minimum allele support of 5 when running MALVIRUS on GISAID assemblies—which we expect to be an usual case.

## Conclusions

In this work, we presented MALVIRUS, an application for analyzing newly-sequenced viral strains. Starting from a read sample and a set of known variations that can be easily produced using MALVIRUS utilities, it allows to genotype the input sample, annotate the genotyped variants, and—in the case of a SARS-CoV-2 virus sample—assign it to the most likely Pango lineages.

As shown by our results, MALVIRUS is able to efficiently and accurately genotype a newly sequenced SARS-CoV-2 virus both from short (Illumina) and long (Oxford Nanopore) reads. Moreover, it also assign the sample to the correct Pango lineage with very high accuracy (40 samples out of 41 in our experimental setting). Finally, MALVIRUS efficiency and accuracy heavily depend on the considered variant catalog. Therefore, we tested different catalogs built with multiple pipelines from different sets of assemblies: from the publicly available assemblies on NCBI/ENA to the more complete but non redistributable assemblies available on GISAID. As expected, the more assemblies are considered for building the catalog, the more information MALVIRUS can use, increasing the quality of the analysis of a new sample, albeit at the expense of its running times.

Since MALVIRUS benefits from comprehensive variant catalogs, the constantly increasing number of available strains will broaden the completeness of the current variant knowledge, thus boosting the overall accuracy of our pipeline.

## Availability and requirements

Project name: MALVIRUS

Project home page: https://algolab.github.io/MALVIRUS

Operating system(s): Platform independent (Docker container)

Programming language: C++ / Python / JavaScript

Other requirements: Docker 20 or higher

License: GNU GPL-3.0

Any restrictions to use by non-academics: none

### Supplementary Information

**Additional file 1:** The full list of samples, laboratories, and authors of the data retrieved from GISAID and used in this manuscript.

## Data Availability

All the programs, scripts, and workflows needed to reproduce the analyses are available in the GitHub MALVIRUS-repro repository: https://github.com/AlgoLab/MALVIRUS-repro/. The datasets analysed in first experimental part (Genotyping accuracy) are available from GenBank and SRA and the aforementioned repository provides detailed instraction to retrieve them. The datasets analysed in the second experimental part also include some data available from GISAID but restrictions apply to redistribution of these data, which were used under license for the current study, and are publicly available from the GISAID website subject to GISAID’s Terms and Conditions (https://www.gisaid.org/registration/terms-of-use/). The aforementioned repository provides detailed instructions to retrieve them. The full list of samples, laboratories and authors of the data retrieved from GISAID and used in this manuscript is available as Additional file 1.

## Abbreviations

SNP: Single-nucleotide polymorphism
VOC: Variant of concern
VOI: Variant of interest
VCF: Variant call format
SRA: Sequence read archive
ILL: Illumina
ONT: Oxford Nanopore Technologies
